# Uncovering nasopharyngeal carcinoma from chronic rhinosinusitis and healthy subjects using routine medical tests via machine learning

**DOI:** 10.1371/journal.pone.0274263

**Published:** 2022-09-09

**Authors:** Qi Liu, Jinyang Du, Yuge Li, Guiyuan Peng, Xuefang Wang, Yong Zhong, Ruxu Du

**Affiliations:** 1 Shien-Ming Wu School of Intelligent Engineering, South China University of Technology, Guangzhou, Guangdong, China; 2 Dept. of Statistics, Chinese University of Hong Kong, Guangzhou, Hong Kong SAR, China; 3 The Second Clinical College of Guangzhou University of Chinese Medicine, Guangzhou, Guangdong, China; University Putra Malaysia, MALAYSIA

## Abstract

Nasopharyngeal carcinoma (NPC) is one of the most common types of cancers in South China and Southeast Asia. Clinical data has shown that early detection is essential for improving treatment effectiveness and survival rate. Unfortunately, because the early symptoms of NPC are rather minor and similar to that of diseases such as Chronic Rhinosinusitis (CRS), early detection is a challenge. This paper proposes using machine learning methods to detect NPC using routine medical test data, namely Random Forest (RF), Support Vector Machine (SVM), and Artificial Neural Network (ANN), k-Nearest-Neighbor (KNN) and Logistic Regression (LR). We collected a dataset containing 523 newly diagnosed NPC patients before treatment, 501 newly diagnosed CRS patients before treatment as well as 600 healthy controls. The routine medical test data including age, gender, blood test features, liver function test features, and urine sediment test features. For comparison, we also used data from Epstein-Barr Virus (EBV) antibody tests, which is a specialized test not included among routine medical tests. In our first test, all four methods were tested on classifying NPC vs CRS vs controls; RF gives the best overall performance. Using only routine medical test data, it gives an accuracy of 83.1%, outperforming LR by 12%. In our second test, using only routine medical test data, when classifying NPC vs non-NPC (i.e. CRS or controls), RF achieves an accuracy of 88.2%. In our third test, when classifying NPC vs. controls, RF using only routine test data achieves an accuracy significantly better than RF using only EBV antibody data. Finally, in our last test, RF trained with NPC vs controls, using routine test data only, continued to perform well on an entirely separate dataset. This is a promising result because preliminary NPC detection using routine medical data is easy and inexpensive to implement. We believe this approach will play an important role in the detection and treatment of NPC in the future.

## Introduction

Nasopharyngeal carcinoma (NPC) is a malignant tumor in nasopharyngeal epithelial cells, with unique geographic and ethnic distributions. It is reported that NPC often occurs in East and Southeast Asia [1], especially in Guangdong, China. The incidence rate is about 25 cases per 100,000 people, which is 25 times higher than in other regions of the world. NPC has posed a serious challenge to public health [2].

The incidence area of NPC is mainly at the top of the nasopharynx and the pharyngeal recesses on both sides. Being sheltered behind other tissues and organs, the location of the lesion is difficult to find. Moreover, early symptoms are not obvious. Therefore, it is difficult to distinguish the onset of NPC from other benign disorders such as sinusitis and rhinitis. By the time it is detected, 70–80% of NPC patients are already in a middle or advanced stage. Through the cervical lymph nodes, NPC may metastasize to distant parts of the body, greatly increasing mortality [3, 4]. At present, the preferred treatment for NPC is radiotherapy, followed by chemotherapy. The prognosis of NPC is closely related to the stage at which it is detected. The consequences of late detection can be fatal. Studies have shown that the 5-year overall survival rate of patients with stage I-II NPC after radiotherapy is as high as 90.4%. For patients with stage III-IV NPC, the 5-year overall survival rate is decreased by more than 15% for each stage [5]. Moreover, systemic and local adverse reactions caused by radiotherapy and chemotherapy, such as radiation-induced oral mucositis, dry mouth, limited mouth opening, cognitive impairment, and sinusitis, etc., will seriously affect the quality of life of patients [6]. Therefore, screening of high-risk groups and early detection is very important.

The pathogenesis of NPC is still unclear. Current research suggests NPC is caused by three categories of factors: Epstein-Barr Virus (EBV) infection [7–9], environmental factors (especially the consumption of Guangdong pickled fish) [10–12], and genetic factors [13, 14]. Of these three, the most relevant to this paper is EBV infection. EBV infected cells express different proteins in the incubation period and the lysis period. In the incubation period, infected cells mainly synthesize core antigen and latent membrane protein; in the lysis period, infected cells primarily synthesize early membrane antigen, early intracellular antigen, and capsid antigen. Therefore, NPC patients have specific antibodies against the EBV. Henle et al [15] found as early as 1976 that the serum of NPC patients has a significantly higher level of EBV antibodies than that of people without NPC. Thus, serological detection of specific antibodies against the EBV is a useful means for detecting NPC. The anti-EBV specific antibodies currently used in clinical nasopharyngeal carcinoma detection include: VCA-IgA, EA-IgA, EBNA-IgA, EBV DNA enzyme antibodies, etc. [16]. The tests for EBV VCA-IgA and EA-IgA are the most common and mature. Cheng et al [17] collected data from 121 newly diagnosed NPC patients before treatment and 332 healthy subjects and found that the sensitivity and specificity of single VCA-IgA were 93% and 87%, respectively. Liu et al [18] evaluated the value of EBV-DNA, EA-IgA, VCA-IgA, EBNA1-IgA, and RTA-IgG in the detection of NPC. Their study included 8382 NPC patients and 15,089 healthy subjects. They found that the sensitivity and specificity of EA-IgA and VCA-IgA were 55%, 96%, 85%, and 89%, respectively. It is worth noting that VCA-IgA has a high detection rate in healthy people; hence its specificity is lower. On the other hand, EA-IgA has strong specificity, but low sensitivity. Consequently, EBV antibody tests are usually not included in routine medical tests and thus most people miss their chance at early detection of NPC.

In recent years, artificial intelligence (AI) has become an important tool for medical diagnosis. As an essential branch of AI, machine learning has been widely used in the construction of medical diagnosis models. Nevertheless, few studies have been conducted thus far to examine the validity of using machine learning in detecting NPC.

It is known that medical data is very complex. It is difficult to find relationships in the data by manual inspection. Machine learning can make full use of complex medical data, finding hidden patterns to achieve more accurate and efficient diagnosis while reducing the workload of doctors. Zou et al [19] used decision trees, Random Forests (RF), and Artificial Neural Network (ANN) to predict diabetes. The RF method performed best, with accuracy, sensitivity and specificity of 89.63%, 92.26%, and 87.00%, respectively. Oh et al [20] proposed to use deep learning network for early detection of Parkinson’s disease. It achieved a promising performance of 88.25% accuracy, 84.71% sensitivity, and 91.77% specificity. Alickovic and Subasi [21] used genetic algorithm-based feature selection to find the most informative features for breast cancer diagnosis, and used different machine learning algorithms to distinguish between benign and malign tumor in breast cancer, including Logistic Regression (LR), Decision Trees, RF, Bayesian Network, Multilayer Perceptron (MLP), Radial Basis Function Networks (RBFN), SVM and Rotation Forest. It is observed that the Rotation Forest achieved the highest classification accuracy of 99.48%. Sharma et al [22] presented a comparative study on the detection of breast cancer using different machine learning algorithms including RF, k-Nearest-Neighbor (KNN) and Naïve Bayes. Their results showed that KNN had the best accuracy, precision and F1 score over the other algorithms. Wen et al [23] indicate that a multi-analyte biomarker panel is clinically useful during health check-ups for the screening of tumors such as hepatocellular carcinoma (HCC) and prostate malignancies. Their biomarker panel consisted of eight molecules: α-fetoprotein, carcinoembryonic antigen, prostate-specific antigen, CA19-9, CA125, CA15-3, squamous cell specific antigen, and cytokeratin 19 fragment. Wang et al [24] combined multiple serum tumor markers to detect various cancers using machine learning methods such as SVM and k-nearest neighbor. They found that these machine-learning methods outperformed the use of individual tumor makers. Wang et al [25] demonstrated machine learning models using many biomarkers are capable of improving early detection of cancer by using a large real world dataset.

The objective of this paper is to study the performance of a selection of machine learning methods for the detection of NPC using routine medical tests. The machine learning methods we use are: Random Forest, Support Vector Machine, Artificial Neural Network and k-Nearest-Neighbor. For comparison with a classical method, we include some performance comparisons with Logistic Regression.

## Materials and methods

### Data collection and processing scheme

The data are collected from two hospitals: the Guangdong Provincial Hospital of Traditional Chinese Medicine–University Town Hospital and the Guangdong Provincial Hospital of Traditional Chinese Medicine–Main Hospital. Our main dataset contains a total of 1624 people recorded in the hospitals from 2013 to 2020 including 523 newly diagnosed NPC patients before treatment, 501 newly diagnosed Chronic Rhinosinusitis (CRS) patients before treatment and 600 healthy controls. The controls were randomly selected from 6873 people who came to the hospitals for routine medical checkups and were found to be free from NPC and other chronic diseases. In addition, we collected a secondary, smaller dataset consisting of 101 newly diagnosed NPC patients prior to treatment, who visited the hospitals between March 1 and Nov 30, 2021, and 100 healthy controls, who visited the hospitals during the same period.

We could identify individual participants during or after data collection.

Diagnosis of CRS followed the Chinese CRS Diagnosis and Treatment Instruction (2021 Kunming version), which is a modified version of an European position paper on rhinosinusitis and nasal polyps (EPOS) [26]. All CRS cases were confirmed by pathology testing.

Diagnosis of NPC follows the TNM staging system, which considers the degree of local invasion of the primary tumor (T), the extent of regional lymph node metastasis (N), and the presence of distant metastasis (M) [27]. The current clinical staging standard is the UICC/AJCC 8^th^ edition / China 2017 edition, as defined below.

- Stage I (TNM classification: T1N0M0): The lesion was confined to the nasopharynx.
- Stage II (TNM classification: T2N1M0): The tumor invaded the surrounding soft tissue and the whole nasal cavity, with single lymph node metastasis less than 6 cm in diameter and above the supraclavicular fossa.
- Stage III (TNM classification: T3N2M0): The tumor invaded the skull base, with bilateral lymph node metastasis less than 6cm in diameter and above the supraclavicular fossa.
- Stage IV (TNM classification: T4N3M0): The tumor invaded the intracranial and cranial nerves and the orbit. Lymph node diameter is greater than 6 cm and there is supraclavicular fossa lymph node metastasis.

All NPC cases were confirmed by pathology testing. The clinical stages of the 523 NPC patients ranged from stage II to IV, of which 40 were stage II, 256 were stage III, and 227 were stage IV. The lack of stage I patients and relatively low number of stage II patients was because NPC is rarely detected early; thus data on early stage patients is scarce.

**Fig 1:**
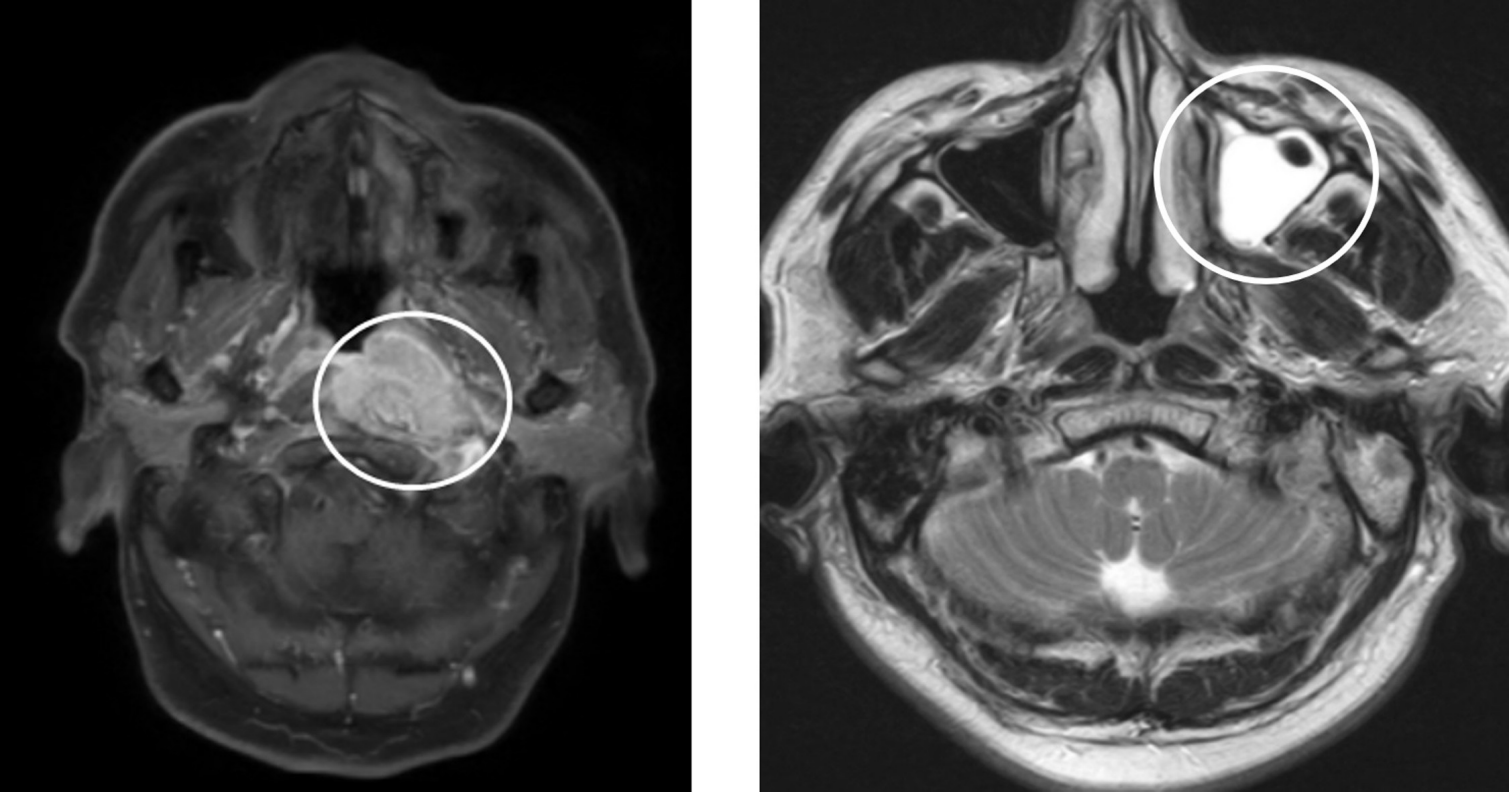
The MRI images. (a) a Stage II NPC patient (b) a CRS patient.

Fig 1A shows the MRI image of a Stage II NPC patient and Fig 1B shows the MRI image of a CRS patient. The question is: Can we distinguish NPC from CRS using routine medical test data.

Each subject in the dataset contains four categories of information, (1) demographic features (gender and age), (2) whole blood test feature indices (testing equipment: Mindray BC-6800 PLUS/6900 Whole Blood Cell analyzer), (3) liver function test feature indices (testing equipment: Roche Cobas 8000 analyzer), and (4) urine sediment test feature indices (testing equipment: Roche U601 semi-automatic urine dry chemistry analyzer). These are all considered routine medical data. Detailed information on the features is shown in Table 1. Note: The number in parentheses are the percentages. It shall be mentioned that some subjects also contained EBV antibody data, specifically VCA-IgA and EA-IgA. It was collected using a YHLO iFlash300-A chemiluminescence analyzer. It was only used in Test 3.

**Table 1:** The subjects’ features.

	NPC Patients	CRS	Controls	*P*-values
**Demographics**				
**Gender**				1.28e-8
Male	403 (77.1%)	307(61.3%)	376 (62.7%)	
Female	120 (22.9%)	194(38.7%)	224 (37.3%)	
**Age**				2.69e-19
19–40 years	118 (22.6%)	203(40.5%)	239 (39.8%)	
41–60 years	273 (52.2%)	217(43.3%)	299 (49.8%)	
>60 years	132 (25.2%)	81(16.2%)	62 (10.3%)	
**Blood test**				
WBC	7.01±2.11	7.04±2.29	6.14±1.17	2.25e-18
NEUT	4.47±1.89	4.45±1.96	3.51±0.92	8.92e-28
LYM	1.77±0.64	2.08±2.06	2.06±0.45	2.22e-5
MONO	0.50±0.20	0.42±0.18	0.37±0.10	4.99e-40
EOSIN	0.21±0.25	0.28±2.06	0.16±0.10	0.21
BASO	0.03±0.02	0.06±0.31	0.03±0.01	0.014
RBC	4.79±1.78	5.36±8.35	4.92±0.36	0.129
Hb	137.38±17.20	141.87±17.78	150.17±11.04	1.05e-41
PLT	262.92±70.55	253.22±61.74	240.59±45.51	2.87e-9
**Liver function test**				
LAP	30.30±48.27	24.88±3.54	24.46±3.55	0.001
ADA	9.96±4.20	8.83±2.59	8.96±2.63	3.45e-9
PA	257.59±57.14	296.50±44.13	296.17±43.12	3.66e-48
ALT	22.41±29.28	20.31±13.79	20.28±8.65	0.112
AST	20.86±17.22	18.82±6.79	20.29±4.07	0.008
GGT	34.13±56.40	27.27±19.83	23.42±10.74	1.27e-6
ALP	78.20±38.49	73.55±23.34	67.38±14.13	1.30e-10
TBIL	9.90±4.07	10.56±10.10	11.10±3.45	0.008
DBIL	3.82±1.59	3.98±3.49	3.94±1.10	0.487
IBIL	6.09±2.85	6.58±9.58	7.14±2.50	0.009
TBA	4.49±6.70	3.39±5.05	2.79±1.64	2.64e-8
Urea	5.31±13.92	5.30±10.75	4.77±1.01	0.582
Cr	82.48±40.83	72.43±16.99	79.07±11.74	3.50e-9
**Urinalysis**				
SG	1.02±0.04	1.02±0.01	1.02±0.01	0.006
PH	5.89±0.64	6.04±0.61	6.13±0.68	5.94e-9
LEU	13.43±69.38	17.94±77.31	6.08±42.30	0.008
PRO	0.02±0.23	0.08±1.54	0.00±0.06	0.275
NTT	0	0	0	\
U_WBC	10.41±87.01	7.06±39.93	1.49±1.32	0.020
Crystal	5.24±24.10	2.43±11.89	0.40±2.81	8.92e-7

In the table, the following abbreviations are used: WBC—white blood cell count; NEUT—Neutrophil count; LYM—Lymphocyte count; MONO—Monocyte count; EOSIN—Eosinophil count; BASO—Basophil count; RBC—Red blood cell count; Hb—Hemoglobin determination; PLT—Platelet count; LAP—Leucine aminopeptidase; ADA—Adenosine deaminase; PA—Prealbumin; ALT—Alanine aminotransferase; AST—Aspartate aminotransferase; GGT— γ-glutamyl transferase; ALP—Alkaline phosphatase; TBIL—Total bilirubin; DBIL—Direct bilirubin; IBIL—Indirect bilirubin; TBA—Total bile acid; Urea—Urea; Cr—Creatinine; SG—Urine Specific Gravity; PH—Urine Ph; LEU—Urinary leukocyte esterase; PRO—Urine protein; NTT—Urine nitrite; U_WBC—Urine white blood cell count; Crystal—Crystal count.

Some data pre-processing was applied. We used Label 0 to represent controls, Label 1 to represent NPC patients, and Label 2 to represent CRS patients. Gender was encoded as a binary variable while age was encoded as an integer-valued variable, accurate to the year. Then, univariate variance analysis was performed to assess the significance of the association between feature indices and the labels. If the *P*-value after Bonferroni correction with a threshold *α* = 0.05 is greater than 0.05, then the feature was deemed insignificant and excluded. Consequently, the following features indices were excluded: EOSIN, RBC, ALT, DBIL, Urea, PRO and NTT. Consequently, a 24-dimensional feature vector was constructed for modeling.

We also considered the problem of missing data. The thirteen features with missing data are: LAP, ADA, PA, ALP, TBIL, DBIL, IBIL, TBA, SG, PH, LEU, U_WBC and Crystal, with missingness rates of 19.3%, 19.3%, 19.2%, 11.9%, 11.9%, 12.7%, 12.9%, 7.7%, 1.8%, 1.7%, 1.7%, 1.7% and 1.7% respectively. These were imputed using an imputation package called “mice” in R using predictive mean matching. Lastly, we normalized all the feature values using the “MinMaxScaler” function in the Python package “sklearn.preprocessing”.

### Methods

We used five machine learning methods: RF, SVM, ANN, LR and KNN. These methods are well-established, but use fundamentally different models and hence provide different views for a problem. The RF was built with Python using the function “RandomForestClassifier” in the package “sklearn.ensemble”. The tree building parameters are searched using the Python function “GridSearchCV” in the package “sklearn.model_selection”. The SVM was built using the function “SVC” in the Python package “sklearn.svm” with the radial basis function kernel function $K(x,x^{\prime })=e^{-\gamma \left\|x-x^{\prime }\right\|}$. The model parameters are searched using “GridSearchCV”. The ANN was built using “Keras” in Python. The network consists of two hidden layers, with 64 nodes in the Hidden Layer 1 and 16 nodes in Hidden Layer 2. We use the rectified linear unit (relu) activation function for both hidden layers. The LR was built with using the function “LogisticRegression” in the package “sklearn.linear_model”. The KNN was built with using the function “KNeighborsClassifier” in the package “sklearn.neighbors”.

For each method, we used the 5-fold stratified cross-validation method to evaluate. First, the dataset was randomly partitioned into 5 subsets of approximately equal size, each with class distribution approximately equal to that of the whole dataset. Then, the union of the 4 subsets was used as the training set and the other subset was used to evaluate the performance. The mean of the five such testing results is used as the outcome. The evaluation indices include: Precision, Recall, Accuracy, Area Under receiver operating characteristic Curve (AUC), and Matthews Correlation Coefficient (MCC).

### Ethics declarations

Ethics approval was obtained from the Ethics Committee of the Guangdong Provincial Hospital of Chinese Medicine (reference number ZE2021-148-01). All research was performed in accordance with the Declaration of Helsinki 2013. This is a retrospective study with a large number of participants. Informed consent was obtained from the participant. In case the participants were not informed and their contact information were lost, waiver of informed consent was approved from the ethics committee.

## Results

A total of four tests were conducted. In Tests 1 and 2, all five methods were tested. For all subsequent tests, we narrowed our attention to RF. Moreover, Tests 1, 2 and 3 used the primary dataset while Test 4 used secondary dataset.

### Test 1—Classification of NPC, CRS and controls

This test was designed to examine whether the machine learning methods could distinguish NPC, CRS and healthy controls as well as which methods performed better.

**Fig 2:**
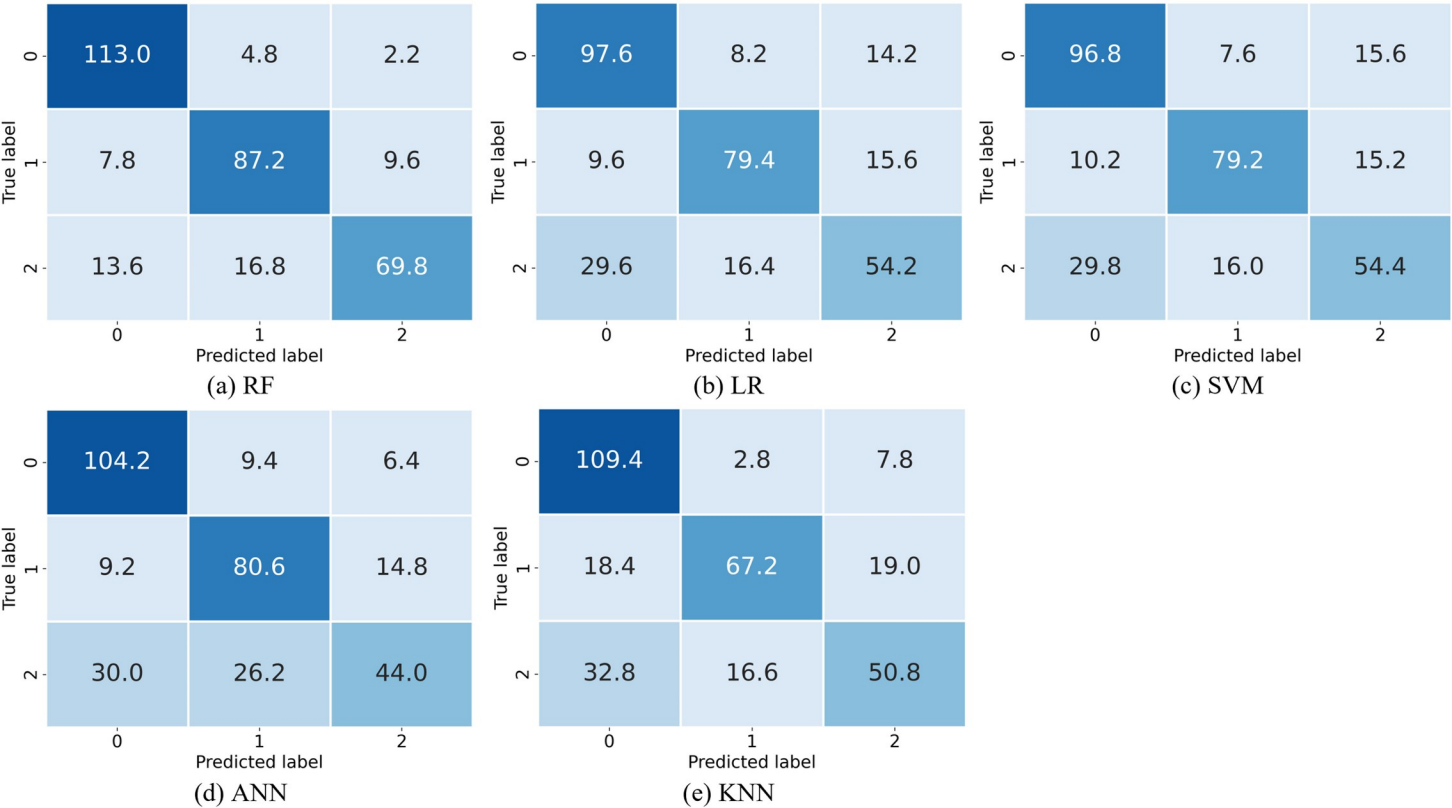
The confusion matrices.

Fig 2 shows the confusion matrices, in which the numbers are the mean of the 5 runs of the 5-fold stratified cross-validation. Table 2 summarizes the performances of the five methods. From the table, it is seen that RF has the best performance achieving 83.1% accuracy with 95% CI above 80%. It also has the best precision, recall and AUC. Moreover, its accuracy outperforms that of the classical LR method by 12%. This indicates that RF is effective.

**Table 2:** The performances of the five machine learning methods.

Method	Precision	Recall	Accuracy	AUC
	(95%CI)	(95%CI)	(95%CI)	(95%CI)
RF	83.3%	82.4%	83.1%	0.954
	(81.5~85.1) %	(76.9~87.9) %	(80.8~85.4) %	(0.947~0.961)
LR	70.8%	70.4%	71.2%	0.874
	(67.6~74.0) %	(63.8~77.1) %	(69.2~73.2) %	(0.865~0.884)
SVM	70.6%	70.2%	70.9%	0.875
	(67.5~73.6) %	(64.0~76.4) %	(69.9~72.0) %	(0.867~0.883)
ANN	70.2%	69.3%	70.5%	0.868
	(68.4~71.9) %	(59.1~79.4) %	(69.7~71.3) %	(0.865~0.871)
KNN	70.5%	68.7%	70.0%	0.864
	(67.4~73.6) %	(59.7~77.7) %	(68.5~71.5) %	(0.855~0.873)

Table 3 shows the performance of RF in more detail. A close examination on Fig 2A reveals that CRS has the most false classification: an average of 13.6 (2.7%) are falsely classified as healthy while an average of 16.8 (3.3%) are falsely classified as NPC. This may be attributed to the fact the CRS is a less severe decease than NPC.

**Table 3:** The performance of RF.

	Precision	Recall	Accuracy	AUC
	(95%CI)	(95%CI)	(95%CI)	(95%CI)
Healthy (Label 0)	84.2%	94.1%		
	(81.3~87.0) %	(91.9~96.4) %		
NPC (Label 1)	80.2%	83.4%		
	(77.2~83.1) %	(78.7~88.0) %		
CRS (Label 2)	85.5%	69.7%		
	(83.6~87.4) %	(67.7~71.7) %		
Average	83.3%	82.4%	83.1%	0.954
	(81.5~85.1) %	(76.9~87.9) %	(80.8~85.4) %	(0.947~0.961)

**Fig 3:**
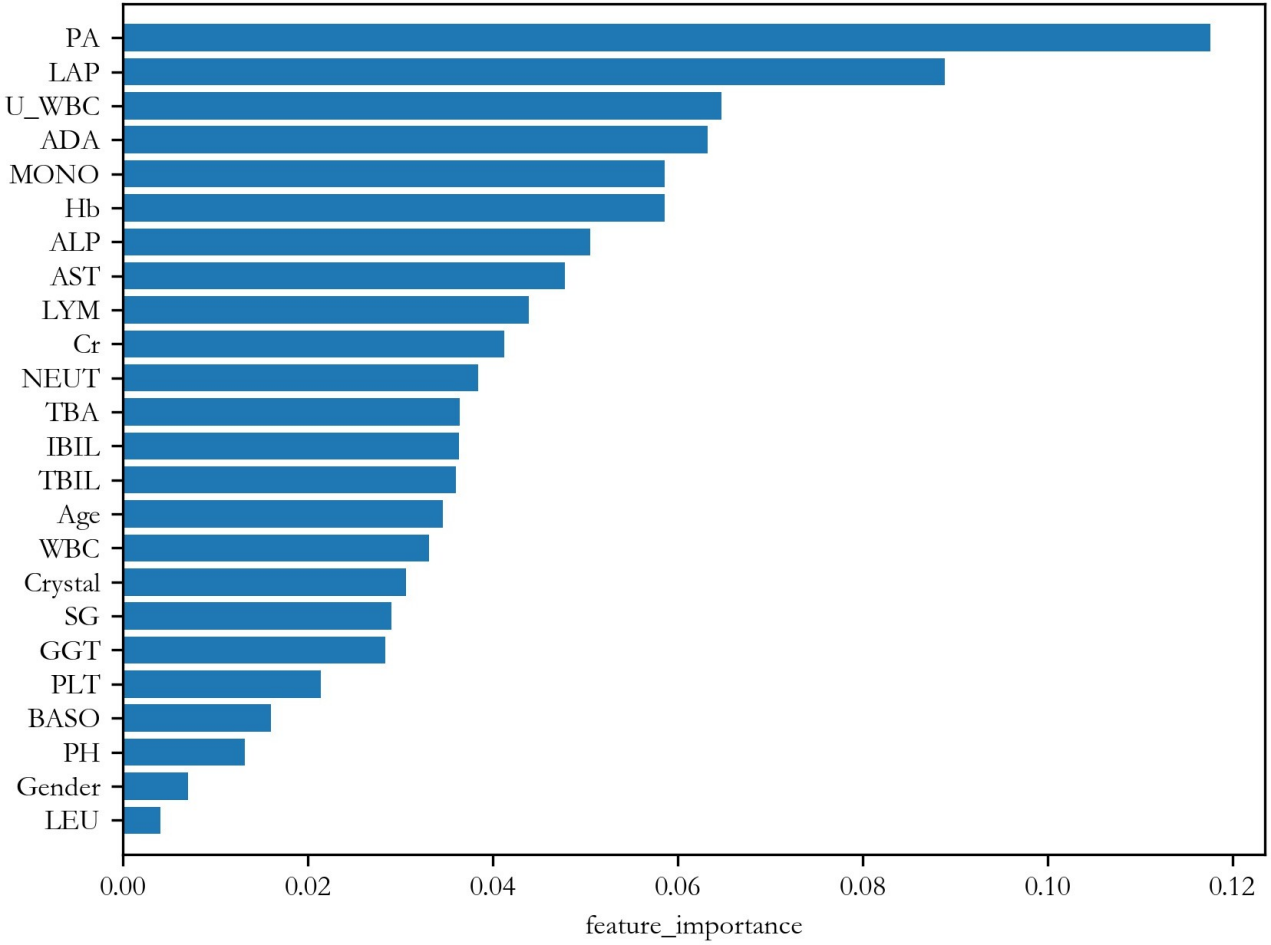
Feature importance ranking from RF.

Fig 3 shows the importance ranking given by RF. The top 8 most important features are PA (weight = 0.118), LAP (0.089), U_WBC (0.065), ADA (0.063), MONO (0.059), Hb (0.059), ALP (0.051) and AST (0.048), respectively. From a biochemical point of view, these feature indices carry important information. For example, PA (prealbumin), LAP (leucine aminopeptidase), ADA (adenosine deaminase), ALP (alkaline phosphatase) and AST (aspartate aminotransferase) are liver function features. Low PA level is a sign of chronic illness and inflammation. MONO (Monocyte) and Hb (hemoglobin determination) are whole blood features indicating immune response. Similarly, U_WBC (urine white blood cell count) is an urine feature related to immune response. As indicated by their weights, none of these feature indices are specific to NPC. However, their combination may reveal some hidden patterns related to NPC.

### Test 2—Detection of NPC

This test is designed to evaluate the ability of machine learning methods in detecting NPC. Two sub-tests are conducted: one is NPC patients vs a mix of 50% of the CRS patients (250 / 501) and 50% of the controls (300 / 600). The other is NPC patients vs CRS patients only.

Table 4 summarizes the performance of five machine learning methods. From the table, it is seen that RF has the best performance achieving 88.2% accuracy again. Hence, we focus on the use of RF in the subsequent discussions.

**Table 4:** Performance of five methods: NPC vs mix of 50% CRS and 50% controls.

Method	Precision	Recall	Accuracy	AUC
	(95%CI)	(95%CI)	(95%CI)	(95%CI)
RF	87.9%	84.7%	88.2%	0.942
	(86.6~89.2) %	(77.9~91.5) %	(86.7~89.7) %	(0.931~0.954)
LR	83.9%	81.7%	85.3%	0.907
	(81.2~86.6) %	(74.8~88.6) %	(83.7~86.9) %	(0.891~0.922)
SVM	83.9%	81.4%	85.2%	0.905
	(81.1~86.7) %	(74.2~88.5) %	(83.3~87.1) %	(0.889~0.922)
ANN	80.5%	78.7%	82.4%	0.886
	(76.5~84.4) %	(71.2~86.3) %	(81.9~82.9) %	(0.872~0.901)
KNN	83.3%	74.6%	82.1%	0.888
	(80.8~85.8) %	(60.9~88.3) %	(80.7~83.5) %	(0.882~0.895)

Table 5 shows the performance of RF in distinguishing NPC patients from CRS patients. From the table, it is seen that the precision in detecting NPC patients is 82.7% and the precision in detecting CRS patients is 85.3%. The AUC value is 0.82.

**Table 5:** Performance of RF: NPC vs CRS.

	Precision	Recall	Accuracy	AUC
	(95%CI)	(95%CI)	(95%CI)	(95%CI)
CRS	85.3%	81.0%		
	(81.6~89.1) %	(78.5~83.5) %		
NPC	82.7%	86.4%		
	(81.2~84.2) %	(82.1~90.7) %		
Average	84.0%	83.7%	83.8%	0.920
	(81.9~86.1) %	(80.8~86.7) %	(81.8~85.7) %	(0.906~0.933)

**Fig 4:**
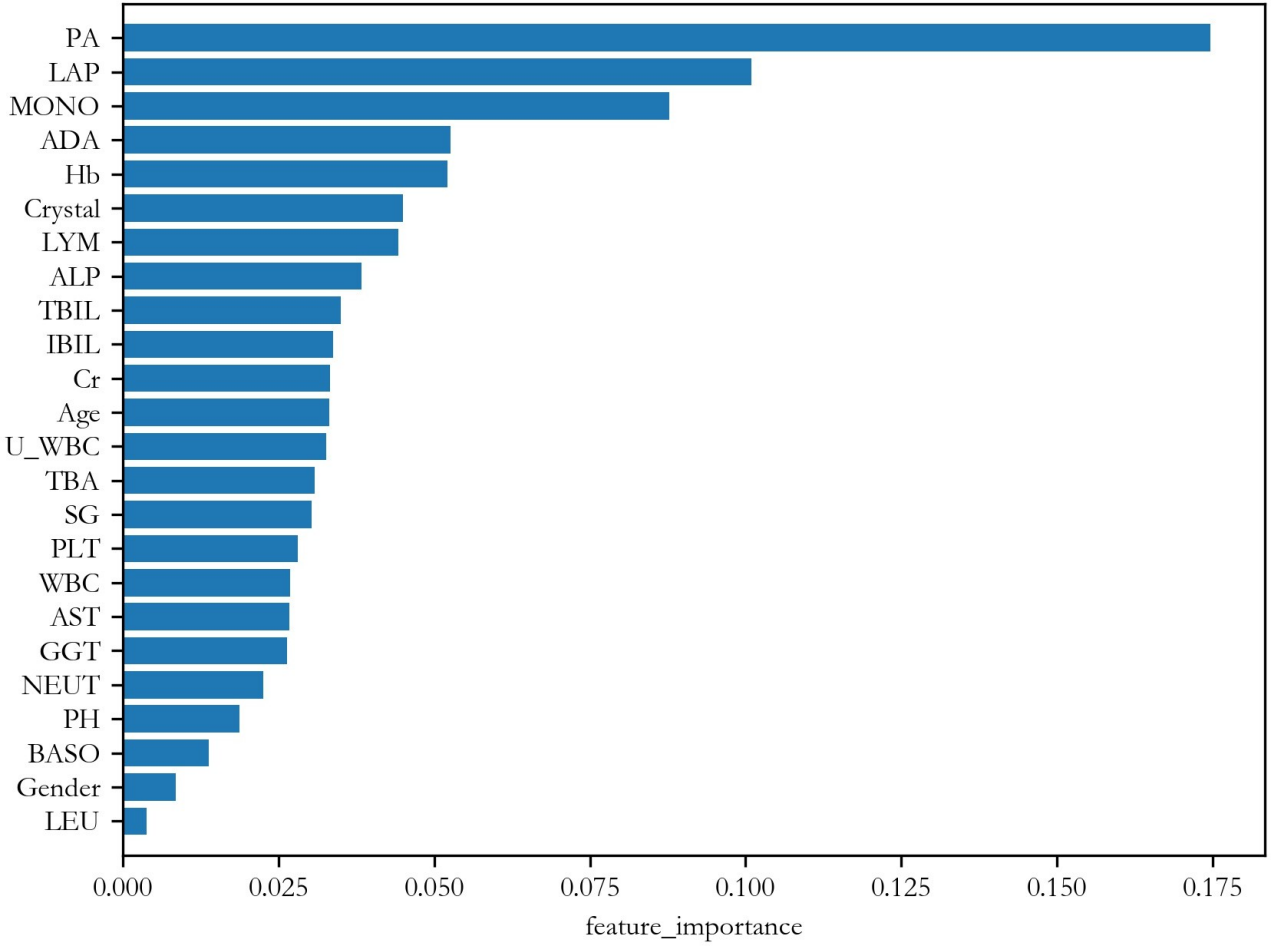
Feature importance ranking when classifying NPC vs. a mixture of 50% CRS and 50% controls.

**Fig 5:**
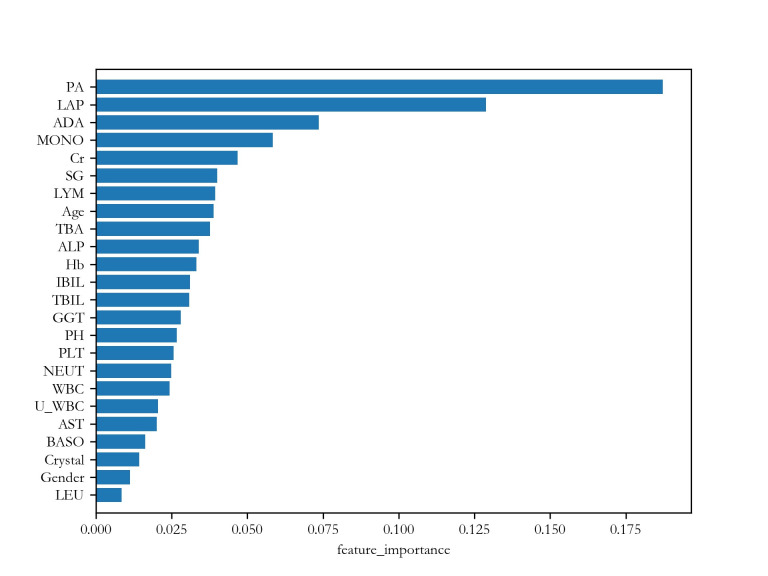
Feature importance ranking when classifying NPC vs. CRS.

Figs 4 and 5 show the importance ranking of the features given by RF. Table 6 lists the top 8 most important features and their weights in these two tests. From the table, it is seen that the first 4 are the same. Moreover, they are also similar to that of Test 1. This is a promising result because it shows that these features are not merely markers of general ill health, but can distinguish NPC specifically from CRS.

**Table 6:** Top importance rankings given by RF.

	NPC vs mixed		NPC vs CRS	
	Feature index	Weight	Feature index	Weight
1	PA	0.175	PA	0.187
2	LAP	0.101	LAP	0.129
3	MONO	0.088	ADA	0.074
4	ADA	0.053	MONO	0.059
5	Hb	0.052	Cr	0.047
6	Crystal	0.045	SG	0.040
7	LYM	0.044	LYM	0.039
8	ALP	0.038	Age	0.039

## Test 3—A comparison to EBV testing

As pointed out in Section 1, EBV antibody tests are effective in detecting NPC. Our third test was to compare the effectiveness of using routine medical test data to detect NPC with the effectiveness of using EBV test data, all with RF. Three sub-tests were conducted in which RF was trained to distinguish NPC patients vs controls; they differed in which features were given to RF: routine medical testing data only, EBV antibody data only, and both. Table 7 shows the classification performances. From the table, we see that RF with routine medical data performs better than RF with EBV data only. Using both routine medical testing and EBV data results in even better accuracy. AUC and MCC figures indicate the same.

**Table 7:** The performance of RF in detecting NPC.

	Use routine medical test only	Use EBV only	Use both
Accuracy (95%CI)	95.0% (93.8~96.2)%	90.4% (87.5~93.2)%	96.9% (95.1~98.7)%
Sensitivity (95%CI)	93.3% (90.3~96.3)%	89.8% (86.9~92.8)%	96.9% (94.5~99.3)%
Specificity (95%CI)	96.5% (94.7~98.4)%	90.8% (87.9~93.8)%	96.8% (95.5~98.2)%
Youden index (95%CI)	89.8% (87.2~92.4)%	80.7% (74.9~86.4)%	93.8% (90.1~97.5)%
AUC (95%CI)	0.986 (0.983~0.989)	0.928 (0.905~0.952)	0.990 (0.982~0.998)
MCC (95%CI)	0.901 (0.877~0.924)	0.807 (0.749~0.864)	0.937 (0.900~0.974)

For this test, we examined the incorrectly classified subjects in more detail. Table 8 shows the number of subjects classified incorrectly and its percentage. False negative cases, i.e. NPC patients classified as healthy, are further broken down by their NPC stage.

**Table 8:** Statistics of misclassified subjects.

		Use routine medical data only	Use EBV only	Use both
False Positive		21 (3.5%)	55 (9.2%)	19 (3.2%)
False Negative	Stage II	3 (7.5%)	3 (7.5%)	2 (5.0%)
	Stage III	18 (7.0%)	30 (11.7%)	9 (3.5%)
	Stage IV	14 (6.2%)	20 (8.8%)	5 (2.2%)
Total		56 (5.0%)	108 (9.6%)	35 (3.1%)

### Test 4 –Testing RF on the secondary dataset

Finally, to evaluate the robustness of RF’s learning, we applied RF, with the forest trained on routine medical features only using the primary dataset, to the secondary dataset. Table 9 shows the results. Recall that the secondary dataset consisted of 101 NPC patients and 100 healthy controls. Tested against this, RF achieved an accuracy of 91.9% (95% CI = 91.5%-92.3%) and the AUC is 0.975. Compared to Test 3 routine medical data only, this is slightly lower (refer to Table 7). This result is consistent with the findings in Test 2 as well (refer to Table 4).

**Table 9:** Performance of RF on the secondary dataset.

	Precision	Recall	Accuracy	AUC
	(95%CI)	(95%CI)	(95%CI)	(95%CI)
Controls	88.2%	96.8%		
	(87.8~88.6) %	(95.7~97.9) %		
NPC	96.5%	87.1%		
	(95.4~97.7) %	(86.5~87.7) %		
Average	92.4%	91.9%	91.9%	0.975
	(89.6~95.2) %	(88.7~95.2) %	(91.5~92.3) %	(0.971~0.979)

## Discussions

In this study, we evaluated the performance of machine learning methods, particularly RF, for detection of NPC. We used a main sample of 1624 subjects, of which 523 were newly diagnosed NPC patients, 501 were newly diagnosed CRS patients and 600 were healthy controls. We also had a secondary sample consisting of 101 NPC patients and 100 controls. In Test 1, we tested the performance of RF, SVM, ANN, LR and KNN at the three-class problem of distinguishing NPC vs CRS vs controls. RF achieved the best performance, with an accuracy of 83.1% and an AUC of 0.954. Furthermore, the most important features as determined by RF were PA, LAP, U_WBC and ADA.

Test 2 consisted of two sub-tests, which tested the performance RF at distinguishing NPC vs a mixture of CRS and controls, and NPC vs only CRS, respectively. In the first sub-test, RF achieved an accuracy of 88.2% and an AUC of 0.942. In the second sub-test, RF achieved an accuracy of 83.8% and an AUC of 0.920. The most important features were PA, LAP, ADA and MONO for the first sub-test and PA, LAP, MONO and ADA for the second sub-test.

Test 3 evaluated the performance of RF at distinguishing NPC vs controls. There were three sub-tests, which differed in which features were given to RF. When RF was given only routine medical data, it achieved an accuracy of 95.0% and an AUC of 0.986. When RF was given only EBV antibodies data, it achieved an accuracy of 90.4% and an AUC of 0.928. When RF was given both, it achieved an accuracy of 96.9% and an AUC of 0.990. This is a promising result because it shows NPC may be accurately detected using only routine medical data, reducing the need for costly EBV tests. The false negative rate of 7.5% for stage II NPC patients is also a promising result because it suggests RF with routine medical data may be effective at detecting even early stage NPC, improving the patient’s chances of survival.

Finally, Test 4 evaluated the performance of the forest trained on routine medical features only in Test 3, applied to classifying subjects in the secondary dataset. It achieved an accuracy of 91.9% and the AUC is 0.975.

We acknowledge some limitations of our study. Our dataset consisted of only 1824 (1624 in the primary dataset and 201 in the secondary dataset) subjects and only 3 classes: NPC, CRS and healthy controls. Ideally, our dataset should not only include a larger total number of subjects, but also a wider variety of health conditions such as other types of cancers. Additionally, the study included no Stage I NPC patient and only 40 Stage II patients. Thus, while the performance of RF at detecting even Stage II NPC patients in Test 3 was promising, a larger study with a more early stage NPC patients is needed to confirm and extend this result.

## Conclusions and future prospects

This paper studies the performance of machine learning methods, particularly Random Forest (RF), at detecting Nasopharyngeal Carcinoma (NPC), using routine medical test data. We believe such methods can play an important role in the future. It can be easily implemented without much additional cost. Its result can serve as a warning; following a positive classification, patients should follow up with a definitive check such as MRI and pathological testing.

Further research should confirm whether they are effective even at detecting early-stage NPC. This idea may also be used to study detection of other types of cancers.

## References

[1] Chen YP, Chan ATC, Le QT, Blanchard P, Sun Y, Ma J. Nasopharyngeal carcinoma. Lancet. 2019;394: 64–80. doi: 10.1016/S0140-6736(19)30956-0 31178151

[2] Chang ET, Adami HO. The enigmatic epidemiology of nasopharyngeal carcinoma. Cancer Epidemiol Biomarkers Prev. 2006;15: 1765–1777. doi: 10.1158/1055-9965.EPI-06-0353 17035381

[3] Chua MLK, Wee JTS, Hui EP, Chan ATC. Nasopharyngeal carcinoma. Lancet. 2016;387: 1012–1024. doi: 10.1016/S0140-6736(15)00055-0 26321262

[4] Perri F, Della Vittoria Scarpati G, Giuliano M, D’Aniello C, Gnoni A, Cavaliere C, et al. Epstein-Barr virus infection and nasopharyngeal carcinoma: The other side of the coin. Anticancer Drugs. 2015;26: 1017–1025. doi: 10.1097/CAD.0000000000000276 26241803

[5] Stenmark MH, McHugh JB, Schipper M, Walline HM, Komarck C, Feng FY, et al. Nonendemic HPV-positive nasopharyngeal carcinoma: Association with poor prognosis. Int J Radiat Oncol Biol Phys. 2014;88: 580–588. doi: 10.1016/j.ijrobp.2013.11.246 24521676 PMC3989890

[6] xia Su Y, ping Liu L, Li L, Li X, juan Cao X, Dong W, et al. Factors influencing the incidence of sinusitis in nasopharyngeal carcinoma patients after intensity-modulated radiation therapy. Eur Arch Oto-Rhino-Laryngology. 2014;271: 3195–3201. doi: 10.1007/s00405-014-3004-8 24659365

[7] Van Doorn M, Vogels J, Tas A, Van Hoogdalem EJ, Burggraaf J, Cohen A, et al. Evaluation of metabolite profiles as biomarkers for the pharmacological effects of thiazolidinediones in type 2 diabetes mellitus patients and healthy volunteers. Br J Clin Pharmacol. 2007;63: 562–574. doi: 10.1111/j.1365-2125.2006.02816.x 17488363 PMC2000756

[8] Fiehn O, Kopka J, Trethewey RN, Willmitzer L. Identification of uncommon plant metabolites based on calculation of elemental compositions using gas chromatography and quadrupole mass spectrometry. Anal Chem. 2000;72: 3573–3580. doi: 10.1021/ac991142i 10952545

[9] Baverel G, Conjard A, Chauvin MF, Vercoutere B, Vittorelli A, Dubourg L, et al. Carbon 13 NMR spectroscopy: A powerful tool for studying renal metabolism. Biochimie. 2003;85: 863–871. doi: 10.1016/j.biochi.2003.10.001 14652175

[10] Fiehn O. Metabolomics—The link between genotypes and phenotypes. Plant Mol Biol. 2002;48: 155–171. doi: 10.1023/A:1013713905833 11860207

[11] Lindon JC, Holmes E, Bollard ME, Stanley EG, Nicholson JK. Metabonomics technologies and their applications in physiological monitoring, drug safety assessment and disease diagnosis. Biomarkers. 2004;9: 1–31. doi: 10.1080/13547500410001668379 15204308

[12] Holmes E, Nicholls AW, Lindon JC, Connor SC, Connelly JC, Haselden JN, et al. Chemometric models for toxicity classification based on NMR spectra of biofluids. Chem Res Toxicol. 2000;13: 471–478. doi: 10.1021/tx990210t 10858320

[13] Brown SC, Kruppa G, Dasseux JL. Metabolomics applications of FT-ICR mass spectrometry. Mass Spectrom Rev. 2005;24: 223–231. doi: 10.1002/mas.20011 15389859

[14] Griffin JL, Kauppinen RA. A metabolomics perspective of human brain tumours. FEBS J. 2007;274: 1132–1139. doi: 10.1111/j.1742-4658.2007.05676.x 17298437

[15] Henle G, Henle W. Epstein‐barr virus‐specific IgA serum antibodies as an outstanding feature of nasopharyngeal carcinoma. Int J Cancer. 1976;17: 1–7. doi: 10.1002/ijc.2910170102 175020

[16] Lo KW, To KF, Huang DP. Focus on nasopharyngeal carcinoma. Cancer Cell. 2004;5: 423–428. doi: 10.1016/s1535-6108(04)00119-9 15144950

[17] Cheng WM, Chan KH, Chen HL, Luo RX, Ng SP, Luk W, et al. Assessing the risk of nasopharyngeal carcinoma on the basis of EBV antibody spectrum. Int J Cancer. 2002;97: 489–492. doi: 10.1002/ijc.1641 11802211

[18] Liu W, Chen G, Gong X, Wang Y, Zheng Y, Liao X, et al. The diagnostic value of EBV-DNA and EBV-related antibodies detection for nasopharyngeal carcinoma: a meta-analysis. Cancer Cell Int. 2021;21: 1–13. doi: 10.1186/s12935-021-01862-7 33691680 PMC7944913

[19] Zou Q, Qu K, Luo Y, Yin D, Ju Y, Tang H. Predicting Diabetes Mellitus With Machine Learning Techniques. Front Genet. 2018;9: 1–10. doi: 10.3389/fgene.2018.00515 30459809 PMC6232260

[20] Oh SL, Hagiwara Y, Raghavendra U, Yuvaraj R, Arunkumar N, Murugappan M, et al. A deep learning approach for Parkinson’s disease diagnosis from EEG signals. Neural Comput Appl. 2020;32: 10927–10933. doi: 10.1007/s00521-018-3689-5

[21] Aličković E, Subasi A. Breast cancer diagnosis using GA feature selection and Rotation Forest. Neural Comput Appl. 2017;28: 753–763. doi: 10.1007/s00521-015-2103-9

[22] Shubham Sharma, Archit Aggarwal TC. Breast Cancer Detection using Machine Learning Algorithms. 2021 9th Int Conf Reliab Infocom Technol Optim (Trends Futur Dir ICRITO 2021. 2021; 114–118. doi: 10.1109/ICRITO51393.2021.9596295

[23] Wen YH, Chang PY, Hsu CM, Wang HY, Chiu CT, Lu JJ. Cancer screening through a multi-analyte serum biomarker panel during health check-up examinations: Results from a 12-year experience. Clin Chim Acta. 2015;450: 273–276. doi: 10.1016/j.cca.2015.09.004 26344337

[24] Wang HY, Hsieh CH, Wen CN, Wen YH, Chen CH, Lu JJ. Cancers screening in an asymptomatic population by using multiple tumour markers. PLoS One. 2016;11: 1–16. doi: 10.1371/journal.pone.0158285 27355357 PMC4927114

[25] Wang HY, Chen CH, Shi S, Chung CR, Wen YH, Wu MH, et al. Improving multi-tumor biomarker health check-up tests with machine learning algorithms. Cancers (Basel). 2020;12: 1–16. doi: 10.3390/cancers12061442 32492934 PMC7352838

[26] Fokkens WJ, Lund VJ, Mullol J, Bachert C, Alobid I, Baroody F, et al. European Position Paper on Rhinosinusitis and Nasal Polyps 2020. Rhinol Suppl. 2012; 1–464. 22764607

[27] Ng WT, Yuen KT, Au KH, Chan OSH, Lee AWM. Staging of nasopharyngeal carcinoma—The past, the present and the future. Oral Oncol. 2014;50: 549–554. doi: 10.1016/j.oraloncology.2013.06.003 23838426

